# Differential expression of hemolysin genes in weakly and strongly hemolytic *Brachyspira hyodysenteriae* strains

**DOI:** 10.1186/s12917-020-02385-5

**Published:** 2020-05-29

**Authors:** Jessica Joerling, Hermann Willems, Christa Ewers, Werner Herbst

**Affiliations:** 1 Institute of Hygiene and Infectious Diseases of Animals, Justus Liebig University Giessen, Frankfurter Str. 85-89, 35392 Giessen, Germany; 2 Department of Veterinary Clinical Sciences, Clinic for Swine, Justus Liebig University Giessen, Frankfurter Str. 112, 35392 Giessen, Germany

**Keywords:** Swine dysentery, *Brachyspira hyodysenteriae*, Hemolysin genes, Transcription, Strong hemolysis, Weak hemolysis, mRNA

## Abstract

**Background:**

Swine dysentery (SD) is a diarrheal disease in fattening pigs that is caused by the strongly hemolytic species *Brachyspira* (*B*.) *hyodysenteriae, B. hampsonii* and *B. suanatina.* As weakly hemolytic *Brachyspira* spp. are considered less virulent or even non-pathogenic, the hemolysin is regarded as an important factor in the pathogenesis of SD. Four hemolysin genes (*tlyA, tlyB, tlyC,* and *hlyA*) and four putative hemolysin genes (*hemolysin*, *hemolysin activation protein*, *hemolysin III*, and *hemolysin channel protein*) have been reported, but their role in strong hemolysis is not entirely clear. Our study aimed to assess the transcriptional activity of eight (putative) hemolysin genes in a strongly hemolytic (B204) and a weakly hemolytic (G423) *B. hyodysenteriae* strain during non-hemolytic and hemolytic growth stages.

**Results:**

Strongly and weakly hemolytic *B. hyodysenteriae* strains caused hemolysis on blood agar at different growth stages, namely during log phase (B204) and stationary/death phase (G423). During the lag, early log, late log (stationary phase in G423) and death phase (time points 1–4) strains differed in their hemolysin gene transcription patterns. At time point 1, transcription of the putative *hemolysin* gene was higher in B204 than in G423. At time point 2, *tlyA* and *tlyC* were upregulated in B204 during hemolysis. *TlyB* and *hlyA* were upregulated in both strains at all time points, but higher transcription rates were observed in the weakly hemolytic strain G423. The transcription activity of the *hemolysin channel protein* gene was quite similar in both strains, whereas the *hemolysin activation protein* gene was upregulated in the non-hemolytic stage of B204 at time point 4. Sequence analysis revealed deletions, insertions and single nucleotide polymorphisms in the G423 *hlyA* promoter, although without altering the transcription activity of this gene.

**Conclusion:**

Our data indicate a combined activity of TlyA and TlyC as the most probable underlying mechanism of strong hemolysis in *B. hyodysenteriae*. Further studies should verify if the expression of *tlyA* is upregulated by the putative *hemolysin* gene. Depending on their immunogenic potential TlyA and TlyC may serve as possible vaccine candidates, especially since vaccines for an effective control of swine dysentery are currently not available.

## Background

Members of the genus *Brachyspira* include fastidious and strictly anaerobic intestinal spirochetes that are divided into strongly and weakly hemolytic species. Strongly hemolytic *Brachyspira* (*B.*) *hyodysenteriae* (shBh), *B. suanatina*, and *B. hampsonii* cause swine dysentery (SD), a globally distributed mucohemorrhagic diarrheal disease in fattening pigs that causes high economic loss, impedes the free trade of animals, and has substantial impact on pig health [1, 2]. Antibiotic treatment is critical for control of SD and is also part of treatment and elimination programs for the disease, especially as no commercial vaccines against SD are available. Currently, pleuromutilins, such as tiamulin, are widely used for this purpose. However, decreased susceptibility of *B. hyodysenteriae* to pleuromutilins has been increasingly reported and in some herds *B. hyodysenteriae* has become resistant to all authorized antimicrobials [2–4]. Antimicrobial resistance could be associated with the multilocus sequence type in *B. hyodysenteriae*, while a correlation with virulence genes including hemolysin genes has not been reported [5, 6].

The strongly hemolytic phenotype of *Brachyspira* spp. is considered the main virulence factor and a key determinant in the pathogenesis of SD [7–9]. Consistent with this, weakly hemolytic *B. hyodysenteriae* (whBh), that were originally identified in 2014, were associated with low virulence in pigs [10–13]. Also the weakly hemolytic species *B. pilosicoli*, *B. intermedia*, and *B. murdochii* cause milder forms of diarrhea or have been considered apathogenic (*B. innocens*) [1].

So far, eight hemolysin genes have been identified in *B. hyodysenteriae*, but only four of them (*tlyA, tlyB, tlyC* and *hlyA*) have been thoroughly examined [14–16]. TlyA is considered a pore-forming hemolysin [14], which can also be found in several other bacteria like *Helicobacter pylori* [17] and *Mycobacterium tuberculosis* [18]. TlyB was found to be homologous to a caseinolytic protease (Clp), while TlyC included a cystathionine beta-synthase (CBS) domain, most likely involved in regulating enzyme activity [15, 19]. Finally, the last phenotypically characterized hemolysin HlyA possibly acts as an acyl-carrier protein (ACP) in the lipid metabolism and is flanked by the genes *fabG* (coding ACP-reductase) and *fabF* (coding ACP-synthase II) [16]. The hemolytic function of the putative hemolysins Hemolysin III, Hemolysin activation protein, Hemolysin channel protein, and Hemolysin, which are encoded by locus tags BHWA1_RS02195, BHWA1_RS02885, BHWA1_RS0905, and BHWA1_RS0470 of the whole genome sequenced *B. hyodysenteriae* strain WA1 (NCBI Reference Sequence NC_012225.1) has been predicted in silico. By comparing their nucleotide sequences with that of hemolysin genes from other bacterial species [20, 21], Hemolysin III and the Hemolysin channel protein [20] were identified as members of the hemolysin III superfamily. In *Bacillus cereus* members of this hemolysin family are considered pore-forming [22]. The Hemolysin activation protein and the putative Hemolysin possess a CBS-domain like TlyC [20].

Studies trying to elucidate the mechanism of strong hemolysis are scarce. Initial experiments with *B. hyodysenteriae* mutants lacking *tylA* yielded clones that showed diminished hemolytic activity on blood agar and reduced virulence in pigs an mice after experimental infection [23, 24]. Transformation of *tlyA*, *tlyB* and *tlyC* genes and their flanking regions into non-hemolytic *E. coli* resulted in a hemolytic phenotype [14], but some authors suggested, that these genes may represent regulatory proteins rather than true hemolysins [16, 25]. However, the induction of hemolysis in *E. coli* by recombinant TlyA of *B. hampsonii* strain 30,446 in a recent study [26] again strengthened its possible role in the strongly hemolytic phenotype of *Brachyspira* spp. Although cloning and sequencing studies also suggested a key role for HlyA in strong hemolysis, its wide distribution among weakly hemolytic *Brachyspira* spp. raised doubts about this [27, 28]. Due to the lack of the putative *hemolysin* gene (*BHWA1_RS04705*) in the weakly hemolytic strain *B. pilosicoli* 95/1000 compared with shBh strain WA1, Wanchanthuek et al. (2010) concluded that this factor might contribute to strong hemolysis [21]. However, Card et al. (2019) recently identified all eight hemolysin genes without any consistent pattern of amino acid (AA) substitution among 34 whBh strains [2].

To sum up, the genetic background of hemolysis in *Brachyspira* spp. is not completely understood. Dynamic transcriptome or proteome studies of the hemolysin genes have not been performed. Therefore, the aim of this study was to assess the role of the eight genes putatively involved in the formation of the strongly and weakly hemolytic phenotype of *B. hyodysenteriae* by measuring their transcriptional activity at different growth phases.

## Results

### Hemolytic phenotype of shBh and whBh and determination of time points for harvesting bacteria for real-time qPCR

**Fig. 1:**
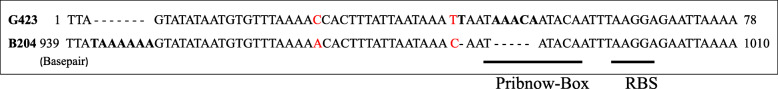
Growth curves (GFU_50_/mL, black line) and hemolytic activity (grey line) of shBh strain B204 in three independent approaches. Arrows indicate the time points where samples were taken for measuring the hemolysin gene transcription

**Fig. 2:**
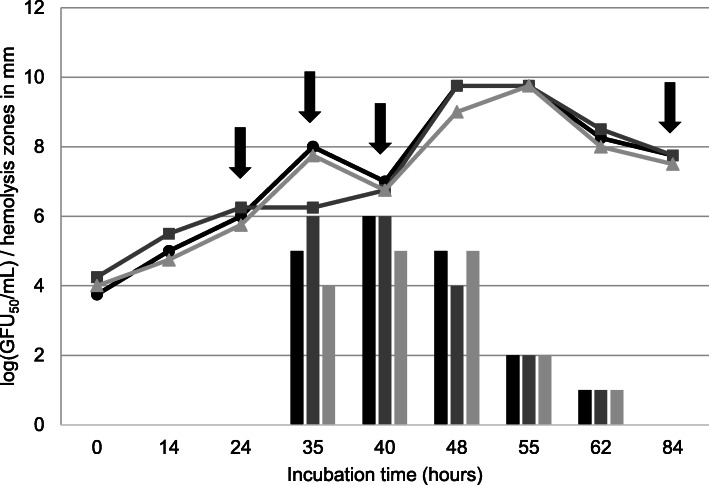
Growth curves (GFU_50_/mL, black line) and hemolytic activity (grey line) of whBh strain G423 in three independent approaches. Arrows indicate the time points where samples were taken for measuring the hemolysin gene transcription

**Fig. 3:**
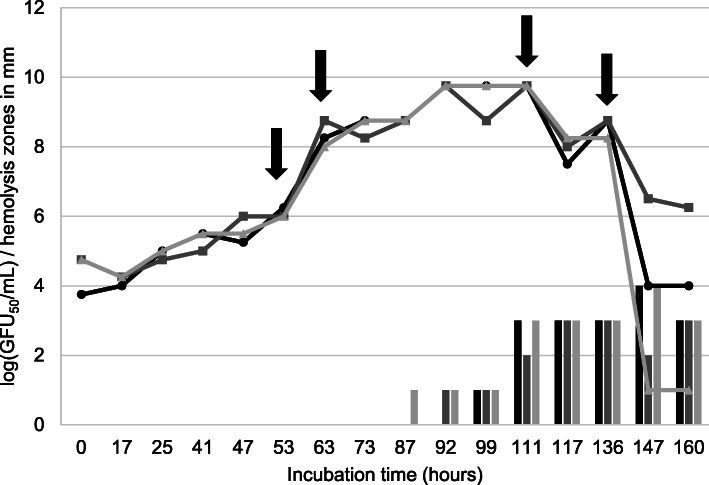
Depiction of hemolysis of culture filtrates of strongly (left) *B. hyodysenteriae* strain B204 (left) and weakly *B. hyodysenteriae* strain G423 (right) by using a hemolysis diffusion test. For semi-quantitative estimation of the hemolytic activity the hemolysis zone was measured (in mm)

The whBh strain G423 revealed much slower growth in BHIF-broth than the shBh strain B204. The strains reached their growth peak, i.e. 10 GFU_50_/mL, at 92 h (G243) and 48 h (B204) after inoculation, respectively. Hemolytic activity was observed after 35 h of incubation for strain B204, and no later than 99 h after incubation (measured in three independent tests) for strain G423. The maximum zone of hemolysis ranged from 3 mm (G423) to 6 mm (B204) (Figs. 1 and 2). The strength of hemolysis (weakly or strong) is illustrated in Fig. 3. Hemolysin gene transcription activity was measured at the following four time points, during the hemolytic and non-hemolytic growth phase of the strains: (i) 24 h (non-hemolytic, lag phase), (ii) 35 h (hemolytic, early log phase), (iii) 40 h (hemolytic, late log phase), and (iv) 84 h (non-hemolytic, death phase) for shBh strain B204 and (i) 53 h (non-hemolytic, lag phase), (ii) 63 h (non-hemolytic, early log phase), (iii) 111 h (hemolytic, stationary phase), and (iv) 136 h (hemolytic, death phase) for whBh strain G423 (Figs. 1 and 2, Table 1). Strain B204 could be sampled before, during, and after hemolysis appeared. This was not possible for strain G423, due to the low population density (GFU_50_/ml), resulting in insufficient amount of mRNA for Real-Time qPCR analysis.

**Table 1:** Live cell count and hemolytic activity of strongly and weakly hemolytic *B. hyodysenteriae* strains during growth in broth and indication of time points chosen for hemolysin gene transcription studies

***B. hyodysenteriae*** strain	Hours of incubation	Hemolysin titer (mm)			Live cell count (GFU_**50**_/mL)			Time point chosen for hemolysin gene transcription study
		during growth in BHIF-broth						
		Trial 1	Trial 2	Trial 3	Trial 1	Trial 2	Trial 3	
B204 (strongly hemolytic)	0	0	0	0	3.75	4.25	4	no
	14	0	0	0	5	5.5	4.75	no
	24	0	0	0	6	6.25	5.75	yes
	35	5	6	4	8	6.25	7.75	yes
	40	6	6	5	7	6.75	6.75	yes
	48	5	4	5	9.75	9.75	9	no
	55	2	2	2	9.75	9.75	9.75	no
	62	1	1	1	8.25	8.5	8	no
	84	0	0	0	7.75	7.75	7.5	yes
G423 (weakly hemolytic)	0	0	0	0	3.75	4.75	4.75	no
	17	0	0	0	4	4.25	4.25	no
	25	0	0	0	5	4.75	5	no
	41	0	0	0	5.5	5	5.5	no
	47	0	0	0	5.25	6	5.5	no
	53	0	0	0	6.25	6	6	yes
	63	0	0	0	8.25	8.5	8	yes
	73	0	0	0	8.5	8.25	8.5	no
	87	0	0	1	8.5	8.5	8.5	no
	92	0	1	1	9.5	9.5	9.5	no
	99	1	1	1	9.5	8.75	9.5	no
	110	3	2	3	9.5	9.5	9.5	yes
	117	3	3	3	7.5	8	8.25	No
	136	3	3	3	8.5	8.5	8.25	yes
	147	4	2	4	4	6.5	1	no
	160	3	3	3	4	6.25	1	no

### Transcription activities of hemolysin genes

**Fig. 4:**
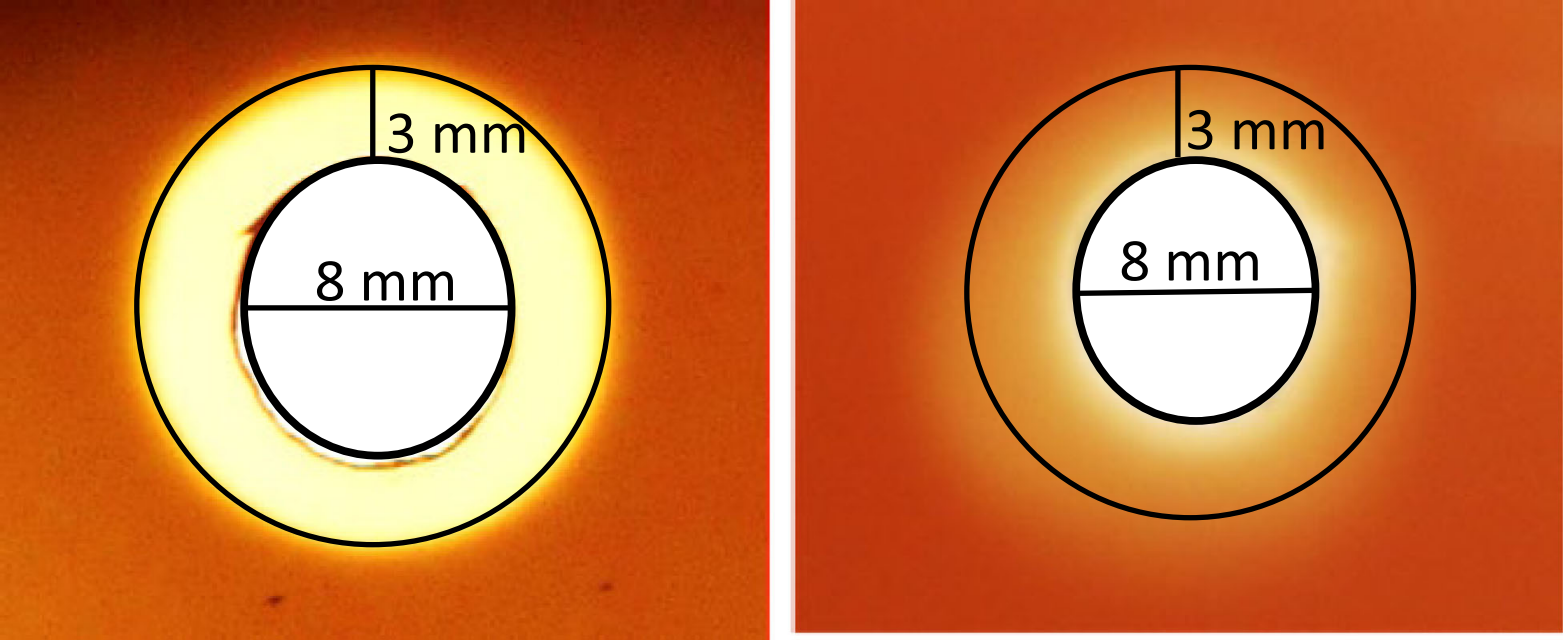
Normalized arithmetic means (three trials) of hemolysin gene qPCR Ct-values from reverse transcribed total RNA prepared from shBh B204 cells at different times of growth

**Fig. 5:**
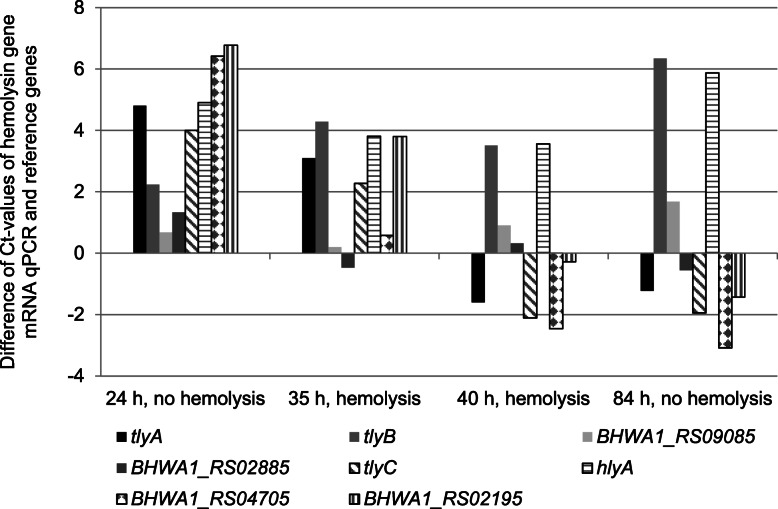
Normalized arithmetic means (three trials) of hemolysin gene qPCR Ct-values from reverse transcribed total RNA prepared from whBh G423 cells at different times of growth

The genes *adh* and *gyrB* served as reference genes for Real-Time qPCR as they were more consistent in standard deviation (StDv) and coefficient of variance (CV) compared with the genes *pgm* and *gdh*. StDv values accounted for 0.37 (*adh*), 0.59 (*gyrB*), 1.63 (*pgm*), and 0.83 (*gdh*) in shBh strain B204 and for 0.71 (*adh*), 0.40 (*gyrB*), 1.21 (*pgm*), and 2.28 (*gdh*) in whBh strain G423. Total RNA concentrations of bacteria ranged from approximately 1.3 ng/μl at the first time point (24 h B204, 53 h G423) to 2.1 ng/μl at the final time point (84 h B204, 136 h G423). Hemolysin genes were differently up- and downregulated in the strongly and weakly *B. hyodysenteriae* strains over time, as shown in Figs. 4 and 5 and in Tables 1 and 2.

**Table 2:** Differences in hemolysin gene transcription activities between weakly hemolytic *B. hyodysenteriae* strain G423 and strongly hemolytic strain B204 determined with the software REST®

Hemolysin gene	Relative expression report of G423 (control strain) compared with B204 (sample strain) determined by the software REST®											
	Time point 1			Time point 2			Time point 3			Time point 4		
	Expression	***p***-value	Result	Expression	***p***-value	Result	Expression	***p***-value	Result	Expression	***p***-value	Result^a^
*tlyA*	1.191	0.795		21.148	0.004	UP	1.403	0.549		0.919	0.881	
*tlyB*	0.490	0.003	DOWN	1.493	0.413		0.168	0.000	DOWN	1.489	0.103	
*tlyC*	1.698	0.380		6.815	0.040	UP	0.288	0.042	DOWN	0.894	0.807	
*hlyA*	0.257	0.146		0.617	0.250		0.035	0.000	DOWN	0.573	0.143	
*hemolysin III*	2.799	0.041	UP	6.269	0.004	UP	0.743	0.485		0.350	0.001	DOWN
*hemolysin activa-tion protein* gene	0.378	0.372		1.234	0.599		2.110	0.050	UP	2.054	0.011	UP
*hemolysin channel protein* gene	0.927	0.873		1.501	0.240		0.490	0.001	DOWN	1.542	0.010	UP
*hemolysin* gene	22.245	0.000	UP	6.088	0.004	UP	1.596	0.173		1.668	0.412	

^a^DOWN = hemolysin gene is downregulated in B204 in comparison with G423 by the mean factor described in the field “expression”, UP = hemolysin gene is upregulated in B204 in comparison with G423 by the mean factor described in the field “expression”

The mRNA transcripts of *tlyA* did not differ significantly among both strains at the first study point (lag phase in both strains, no hemolysis). At the second time point (early log phase in both strains, B204 hemolysis) *tlyA* of B204 was 21-fold upregulated compared to whBh strain G423 (*p*-value 0.004). At the third (B204 late log phase, G423 stationary phase, both strains hemolysis) and fourth study point (death phase in both strains, G423 hemolysis) the transcription activity of *tlyA* differed only marginally between both strains (*p*-value > 0.05). *TlyC* showed a quiet similar transcription pattern as *tlyA*, as apparent from a 6.8-fold upregulation in strain B204 at the second time point, where only B204 showed hemolytic activity (*p*-value 0.04). Except for time point 3, where *tlyC* was downregulated in the shBh strain, remarkable differences in the transcription activities of the strains were not detected.

*HlyA* and *tlyB* were upregulated in both strains at all four time points. Upregulation was slightly higher in strain G423 than in strain B204 and became significant at time point 3 for *hlyA* and *tlyB* (*p*-value < 0.001) and at time point 1 for *tlyB* (*p*-value 0.003). The *hemolysin III* gene (*BHWA1_RS02195*) was upregulated in both strains (no hemolysis) at the first time point. Upregulation was 2.8-fold higher in B204 (*p*-value 0.041) compared with G423. At the second time point (B204 hemolysis), the transcription of *hemolysin III* was increased by 6.3-fold in the shBh strain B204 (*p*-value 0.004). At the third point (both strains hemolysis) the transcription activity of *hemolysin III* gene was almost identical in both strains. At time point four (G423 hemolysis) *hemolysin III* was significantly downregulated in strain B204 (*p*-value 0.001).

The *hemolysin activation protein* gene (*BHWA1_RS02885*) was upregulated (*p*-value > 0.05) at the first time point in both strains. During the third and fourth time point transcription increased by 100% in the shBh B204. During the lag phase the *hemolysin* gene (*BHWA1_RS04705*) was initially upregulated 22-fold in B204 compared with G423 (*p*-value < 0.001). At the second time point *hemolysin gene* transcription was 6-fold higher in B204 compared with G423. During the third and fourth time point significant differences between both strains were not observed (*p*-value > 0.05). Finally, the transcription activity of the *hemolysin channel protein* gene (*BHWA1_RS09085*) was almost comparable to that of the reference genes in both strains. Only at the third time point (onset of weak hemolysis) this gene was significantly downregulated in the shBh strain B204 (*p*-value 0.001). In addition, a slight upregulation (1.5-fold) was identified in the shBh strain B204 at the final study time point.

### Sequence alignments of different gene fragments of whBh strain G423

**Fig. 6:**
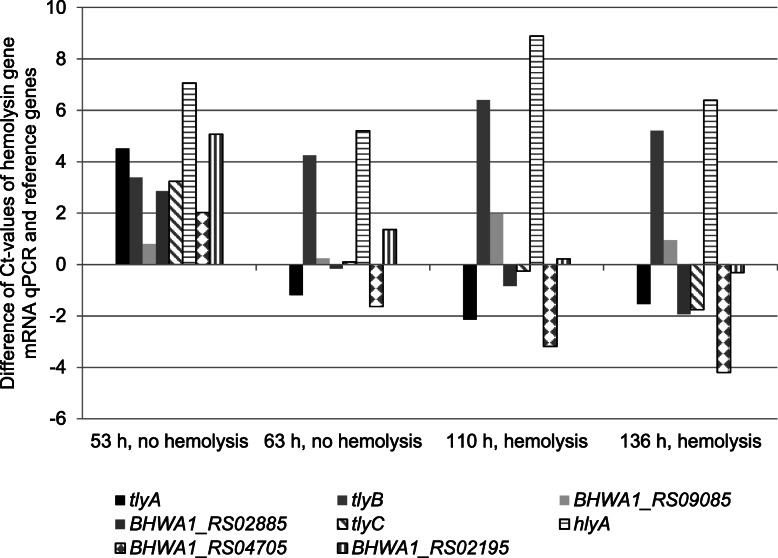
Alignment of the deduced *hlyA* promoter site nucleotide sequence of whBh G423 to the corresponding sequence of whBh strain B204 (Accession no. U94886, pos. 939–1010). Single nucleotide polymorphisms are underlined and printed in grey. The Pribnow box as well as the ribosome binding site (RBS) are underlined in bold

The nucleotide sequences of the *nox* gene fragment (939 bp) and the 16S rRNA gene fragment (870 bp) of whBh strains G423 were 99% identical to the genes in *B. hyodysenteriae* strain 49 (GenBank accession no. KU215621 and KU215620) and *B. hyodysenteriae* strain ATCC 27164 (NCBI Reference Sequence NZ_CP015910.2), respectively. Compared with shBh strain B204, strain G423 revealed deletions, insertions and mutations in the presumed promoter site of *hlyA* (Fig. 6). In detail, the G423 *hlyA* fragment had a length of 71 instead of 72 bp (B204), revealed two single nucleotide polymorphisms at position 30 (g.30A > C) and 46 (g.46C > T), a deletion of seven base pairs starting at position 4 (g.4_10delTAAAAAA), one single nucleotide insertion at position 47 (g.47insT), and an additional insertion of 5 nucleotides at position 52 (g.52_56insAAACA). Mutations at the presumed ribosome binding site were not observed [11]. The nucleic acid sequence of G423 differed by 19.2% from that of B204.

## Discussion

The aim of this study was to provide insight into the as yet only partially resolved mechanism of strong hemolysis in *B. hyodysenteriae* by comparing the transcriptional and hemolytic activity of a shBh and a whBh strain at different growth stages. To date, weakly hemolytic *B. hyodysenteriae* strains have been solely recovered from pigs without clinical signs of swine dysentery [11, 13]. This strengthens the role of strong hemolysis in the pathogenesis of SD and points out the necessity to combine molecular and phenotypical methods for characterization of *Brachyspira* spp. strains [2].

So far, neither genomic nor proteomic analyses of *Brachyspira* spp. could clearly verify the mechanism of strong hemolysis [2, 11, 13, 29]. Dynamic studies investigating *B. hyodysenteriae* hemolysin expression profiles at the hemolytic and non-hemolytic growth stage have not been performed. On the other hand, by deducing a hemolytic phenotype merely from the sequence of putative hemolysin genes, possible transcriptional and translational events and their effect on the phenotype are not appropriately taken into account. In a recent study 41 AA substitutions among the eight hemolysins were deduced from the genome sequences of 34 whBh strains [2]. These mutations were not observed among the genomes of 81 shBh strains. Each of the 34 whBh strains showed AA substitutions in at least one of the putative hemolysins. The maximum number of 16 AA substitutions was found in the hemolysin TlyB, possibly due to higher length of this hemolysin (828 AA) compared to the others. However, only seven of 34 strains revealed AA substitutions in TlyB. In case of hemolysins HlyA, TlyA, and TlyC, and the putative Hemolysin, AA substitutions were identified at one to three positions, respectively. Hemolysin III, Hemolysin activation protein, and Hemolysin channel protein revealed AA substitutions at eight (Hemolysin III), seven (Hemolysin activation protein) or four (Hemolysin channel protein) positions. Due to the lack of structural protein data, the activity of the putative proteins could not be predicted. As none of the mutations consistently occurred in all 34 whBh strains, a clear association with the lack of strong hemolysis could not be determined. Although the analysis of hemolysins did not reveal a consistent pattern of AA substitutions among the whBh strains, results from the MLST indicated that the whBh strains form a distinct sub-clade within the species *B. hyodysenteriae*. The authors suggested that the formation of this sub-clade may have arisen from an adaption of the bacteria to antimicrobial therapy and that the reduced virulence of whBh may confer the pathogen the ability to persist in its host [2].

In our study, we compared the hemolysin gene transcription rates of whBh strain G423, which was isolated from a pig suffering from mild diarrhea in our routine veterinary microbiological diagnostic laboratory in 2014, and shBh strain B204. We could show that the whBh strain G423 releases its hemolysin(s) at the stationary and the death phase of growth. In contrast, hemolysis in the shBh strain B204 occurred during the logarithmic phase only. This corresponds well with previous observations about the time-dependent hemolytic activity of strongly and weakly hemolytic *Brachyspira* spp. on solid media, i.e. on blood agar plates [30]. Our results differ from early studies of Lemcke et al. [31] who showed that the highest hemolysin titer (128) of shBh strain P18A grown in liquid broth appeared in the stationary phase (72 h). Phenotypic studies to determine hemolysis titers in whBh strains are yet not available. Consequently, the time point of hemolysin release and timely development of hemolysin titers could not be predicted. *B. pilosicoli* strain PWS/B, another weakly hemolytic species, exhibited the highest hemolysin titer (256) after 30 h of incubation in the early logarithmic phase [31]. As the reason for the different onset of hemolysis is yet unclear, we took samples to determine the hemolysin gene transcription activities before, during, and, if possible, after the hemolytic stage in our strains. In contrast to the development of hemolysis, the growth curves (GFU_50_/mL) of both *B. hyodysenteriae* strains showed only slight differences, presumably due to the slower growth of the whBh strain G423.

To normalize the hemolysin gene transcription activity, we used *gyrB* as reference gene following the protocol of Witchell et al. [32]. In that study, transcription activities of the *B. hyodysenteriae* outer membrane protein genes *bhmp39a-h* were investigated. Since multiple reference genes have been recommended for the normalization of Ct-values [33], we included *adh* as a second reference gene in our study. Furthermore, in order to consider the possibility of co-regulation, the reference genes should belong to different functional classes [33]. In our case, *adh* belongs to class 1 oxidoreductases and *gyrB* belongs to class 5 isomerases (https://enzyme.expasy.org/enzyme-byclass.html).

We observed few, but significant differences between the hemolysin gene transcription patterns of *B. hyodysenteriae* strains B204 and G423. During the lag phase (first time point) the *hemolysin* gene was 22-fold upregulated in shBh strain B204 compared with whBh strain G423. It may be assumed, that the Hemolysin regulates the transcription of hemolysin genes responsible for the strong hemolysis. Although the time from gene transcription to protein translation and possible post-transcriptional modifications of genes in *Brachyspira* species is unknown and may differ from one gene to another, it seems likely that a possible initiator becomes active before the hemolytic phenotype changes. As *tlyA* and *tlyC* were significantly upregulated (*p*-value < 0.05) during early log phase of growth (second time point) in the shBh strain B204, these hemolysin genes may have been triggered by the Hemolysin. Nevertheless, other, so far unrecognized factors might likewise be involved in regulation of *tlyA* and *tlyC*. At the late log and death phase of growth, the amount of *tlyA* and *tlyC* mRNA from strain B204 was below that of the reference genes *adh* and *gyrB*. At this stage, the hemolytic activity of B204 declined or even disappeared (Fig. 4). In previous studies, *tlyA* has already been considered as major player in strong hemolysis, since knock-out mutants were significantly reduced in their hemolytic activity [23, 24]. TlyA as well as TlyB, TlyC*,* and HlyA have been shown to confer a beta-hemolytic phenotype when transferred to non-hemolytic *E. coli* strains [15, 16]. In addition, recombinant TlyA of *B. hampsonii* showed hemolytic activity in *E. coli* [26].

Interestingly, *tlyB* and *hlyA* were higher transcribed than the reference genes throughout the experiment in both strains. During the first (*tlyB*) and third time point (*tlyB*, *hlyA*) whBh strain G423 showed a significant upregulation of these genes compared with strain B204. This finding partially corresponds to results from proteome analysis conducted with different shBh strains. Here, TlyB has been determined as the most abundant hemolysin in *B. hyodysenteriae* [29]. The finding of high transcription rates for *hlyA* and *tlyB* during the entire study period may indicate that an accumulation of the gene products may be required to reach biologic active hemolysin concentrations. This might also explain the late onset of weak hemolysis at the stationary growth phase. The lower transcription rates of *tlyB* and *hlyA* in strain B204 compared to strain G423 remain unclear. The additional presence of a weak hemolysin in shBh strains could be hypothesized. Indeed, we observed of a zone of weak hemolysis that occasionally surrounded the strong hemolytic area of shBh strains in the hemolysin diffusion assay (data not shown). On the other hand, one might assume that *tlyB* and *hlyA* do not represent hemolysin genes but genes that are essential for bacterial growth in liquid media especially in the whBh strain. In a recent study with 31 whBh strains, only few isolates revealed mutations in the hemolysin genes *tlyB* (*n* = 2/31) and *hlyA* (*n* = 7/31) [2]. If these changes could account for the weakly hemolytic phenotype needs to be verified.

Of the putative hemolysin genes, *hemolysin III* was slightly higher transcribed in shBh strain B204 during the first and second time point. If *hemolysin III* truly represents a hemolysin gene is not clear. It’s transcription was heavily impaired as it was disrupted into two parts and separated by a nucleotide sequence of 570.925 bp in length in the strongly hemolytic *B. hampsonii* strain 30,599 [26]. Furthermore, 112 bp of the *hemolysin III* gene were deleted. However, among 20 completely sequenced *B. hyodysenteriae* strains [34] this disruption was not present (in silico analysis, data not shown). Differences in the nucleotide sequences of putative *hemolysin channel protein* gene and *hemolysin activation protein* gene could not be correlated with the presence or absence of hemolysis in the strains. Therefore, these genes may play a negligible role in the formation of strong hemolysis.

In contrast to our findings based on transcriptional data, the *hlyA* gene was formerly hypothesized as major player in strong hemolysis of *B. hyodysenteriae* [13, 16]. Mutations, including nucleotide substitutions, insertions and deletions in the potential promoter region of this gene in the whBh strain JR11 were discussed as a possible reason for the absence of strong hemolysis [11]. Although we identified similar mutations in whBh strain G423, they obviously had no inhibitory effect on the transcription rate of *hlyA* in our study. In contrast, transcription rates were even higher in whBh strain G423*.* Thus, it needs further verification if the reported region represents the promoter of *hlyA* as postulated by La et al. [12]. Because strongly hemolytic activity was not linked to *hlyA* transcription in our study, it is questionable if *hlyA* could cause the strongly hemolytic phenotype without any (unknown) co-factor. HlyA was first identified through N-terminal sequencing of purified hemolysin from the supernatant of *B. hyodysenteriae*. Screening of the *B. hyodysenteriae* genomic library resulted in eight positive plaques and allowed the preparation of recombinant plasmids that caused a hemolytic phenotype in non-hemolytic *E. coli* strains after transformation. However, plasmids contained the two ORFs *fabF* and *fabG* adjacent to *hlyA*, which might contribute to the hemolytic phenotype as well [16]. In addition, changes in the promoter site of *hlyA* are obviously not unique to whBh strains [2]. Instead, Card et al. (2019) identified differences within the promoter sites of *tlyB*, *hemolysin III*, and *hemolysin activation protein* gene that were unique to 34 whBh strains only.

Further studies, including knock-out experiments or transformation studies with the hemolysin genes and their promoter sites may be helpful to clarify the distinct role of each of the eight genes in the hemolytic phenotype, although it must be said that manipulating techniques in the genus *Brachyspira* are methodologically demanding [24, 35]. To verify the existence of a common mechanism of strong hemolysis in all *Brachyspira* spp., additional hemolysin gene transcription studies involving the strong hemolytic species *B. suanatina* and *B. hampsonii* should be performed. In addition, proteomic studies at different time points of the growth of weakly and strongly hemolytic *Brachyspira* spp. are required to clarify possible translational effects or to ensure if the hemolysin genes are translated at all.

## Conclusion

Our data indicate a combined activity of TlyA and TlyC as the most likely mechanism underlying the strong hemolysis in *B. hyodysenteriae*. Depending on the immunogenic potential of TlyA and TlyC, these proteins could serve as possible candidates for a subunit vaccine. If the putative *hemolysin* gene might also contribute to strong hemolysis, either individually, in combination with other genes, or as regulator, requires further investigation. Weak hemolysis might be the result of moderate upregulation of *tlyB* and *hlyA* during the entire growth phase of the bacteria. Slow accumulation of hemolysins over time might explain the late phenotypic manifestation of weak hemolysis in the stationary growth phase.

Resistance of *B. hyodysenteriae* to pleuromutilins and other authorized antimicrobials, such as lincosamides and tylosin, is critical for successful antimicrobial therapy of the infection. Thus, novel strategies for an effective control of SD, probably involving hemolysin genes as targets, are urgently required. Several attempts to develop a vaccine against *B. hyodysenteriae* have failed [36–38] or are still under progress [39]. The hemolysins TlyA and TlyC may be promising candidates for the development of a subunit vaccine to control swine dysentery in pigs.

## Methods

### Brachyspira hyodysenteriae strains, cultivation and purity control

The shBh strain B204 [30] and the whBh strain (G423) were used in this study. Strain B204 was isolated from a pig with SD in the United States of America in the 1970s and strain G423 was isolated during routine microbiology diagnostics from growing pigs suffering from mild diarrhea in Germany in 2014. By using a species-specific PCR that targets the *nox* gene region (amplicon 435 bp, [40]) both strains were confirmed as *B. hyodysenteriae*. Furthermore, examination of hemolysin genes (*tlyA, tlyB, tlyC*, and *hlyA*) and putative hemolysin genes (*hemolysin III* gene, *hemolysin activation protein* gene, *hemolysin channel protein* gene*,* and *hemolysin* gene) showed that these eight genes were present in both strains (data not shown).

Bacteria were cultured on trypticase soy agar (TSA) plates containing 5% (v/v) sheep blood under anaerobic conditions (adapted and modified from Kunkle and Kinyon, [41]). Brain-heart-infusion broth (BHI; Oxoid, Wesel, Germany) containing 20% fetal calf serum (FCS; Biochrom, Berlin, Germany) was used as liquid culture (BHIF). The purity of both strains was confirmed phenotypically based on their typical morphology and by use of dark-field and phase-contrast microscopy (Leica DMR HC microscope, Leitz, Wetzlar, Germany). For the transcription studies (see below), 100 μl-samples were taken every day and were additionally cultured on blood agar under aerobic and anaerobic conditions to exclude bacterial contaminations.

### Experimental setup, determination of hemolytic activity and calculation of growth forming units (GFU_50_/mL) for the hemolysin gene transcription study

BHIF (BHI containing 20% FCS) broth (50 mL) was inoculated with 1 mL *B. hyodysenteriae* B204 and G423, respectively, each containing 3 × 10^5^ live cells, and incubated anaerobically at 37 °C on a shaker. Each experiment was performed in triplicate. At four different growth points (Figs. 1 and 2) samples were taken to determine the hemolytic activity of the strains, the live cell count and hemolysin gene transcription rates. Hemolytic activity in different cell-free supernatants was determined by using an agar diffusion assay. TSA agar was poured into petri dishes and wells of 8 mm in diameter were prepared in the agar and filled with the culture filtrates. After incubation of the plates for 48 h at 37 °C, hemolysis was identified by the presence of a transparent zone surrounding the wells. For semi-quantitative estimation of the hemolytic activity the hemolysis zone sizes were assessed. To calculate GFU_50_/mL series dilutions (on a logarithmic scale) the *B. hyodysenteriae* suspensions were prepared in BHIF at different growth points. Four 10 μL spots from each dilution were given on TSA agar plates. After five days of anaerobic incubation (37 °C) the number of hemolytic spots was used to calculate the titer in GFU_50_/mL according to the formula of Spearman [42] and Kaerber [43]. The four time points chosen for hemolysin gene transcription activity were based on the results from the hemolysin diffusion assay and growth curves (GFU_50_/mL).

### RNA isolation and purification

Total RNA was prepared from samples taken at hemolytic and non-hemolytic stages of growth by use of the RNAprotect Bacteria Reagent (Qiagen, Hilden, Germany). Briefly, 500 μL of cultured bacteria were added to 1000 μL RNAprotect, immediately vortexed, incubated for five minutes at 20 °C, and centrifuged (10 min, 5000 x g, 20 °C). After carefully removing the supernatant, the pellet containing bacterial cells with stabilized RNA was frozen at − 70 °C until further processing. RNA from bacterial cells was extracted with the RNeasy Mini Kit (Qiagen, Hilden, Germany). For this purpose, bacterial cells were suspended in 700 μL lysis buffer, mixed with 500 μL 99% ethanol (Merck KGaA, Darmstadt, Germany) and transferred to a spin column. After centrifugation at 7000 x g for 15 s, wash buffer was added before digestion of DNA with 2 μl DNAseI (1500 Kunitz units; Qiagen, Hilden, Germany) for 15 min at 20 °C. After three washing steps mRNA was eluted with RNase-free water and finally treated a second time with 5 μl DNase (1 U/μL; Thermo Fisher Scientific, St. Leon-Rot, Germany) for 20 min at 37 °C. To protect the RNA from degradation, a total of 3.5 μL RNase inhibitor (20 U/μL, Thermo Fisher Scientific, St. Leon-Rot, Germany) was added to all solutions containing RNA. The concentration of RNA was measured with the Nano Drop 2000C photometer (Thermo Fisher Scientific, St. Leon-Rot, Germany) and the purity was evaluated through the A_260_/A_280_ ratio. RNA was stored at − 70 °C until further usage.

### Reverse transcription

Complementary DNA (cDNA) was generated from purified mRNA by use of forward and reverse primers (Additional file 1). All primers used in this study were checked for their in silico binding activity to the nucleotide sequences of *B. hyodysenteriae* strains B204 (NCBI Reference Sequence no. NZ_JXND00000000) and G423. Nucleotide sequence alignments with sequences deposited in GenBank were performed with BLAST (http://blast.ncbi.nlm.nih.gov/Blast.cgi) and the Software Geneious version 8.1.9 (Biomatters, Auckland, New Zealand).

Each reaction mixture (26 μL total volume) contained 10 μL purified mRNA, 1 μL of each primer (20 μM), 2 μL DTT (0.1 mM Dithiothreitol, Roth, Karlsruhe, Germany), 1 μL MgCl_2_ (50 mM, PAN Systems, Aidenbach, Germany), 4 μL nucleotides (each nucleotide 4 mM, Rapidozym, Berlin, Germany), 5 μL of 5 x buffer (750 mM Tris, 500 mM KCl and RNase-free water), 0.2 μL RNase Inhibitor (20 U/μL, Thermo Fisher Scientific, St. Leon-Rot, Germany), and 0.3 μL MultiScribe Reverse Transcriptase (50 U/μL, Thermo Fisher Scientific, St. Leon-Rot, Germany). After incubation at 40 °C for 42 min samples were stored at − 20 °C until further usage.

### Reference genes for real-time quantitative PCR

Three genes (*adh*, *pgm*, *gdh*), that are also used in the 7-MLST scheme of *B. hyodysenteriae*, and the gyrase gene *gyrB* gene, which was already used in a transcription study by Witchell et al. [32], were compared by using the Excel-based software BestKeeper version 1 (https://www.gene-quantification.de/bestkeeper.html) to identify the two most appropriate reference genes for this study [44]. This software computed a descriptive analysis for each reference gene, including geometric and arithmetic mean, minimal and maximal value, standard deviation (StDv) as well as the coefficient of variance (CV). To estimate the inter-gene relations of reference genes numerous pair-wise correlation analyses were performed.

### Verification of absence of genomic DNA

Absence of genomic DNA was verified by conventional PCR with primers targeting the hemolysin and reference genes. Each reaction mixture (30 μL total volume) contained 1 U PanScript DNA Polymerase (PAN Systems, Aidenbach, Germany), 0.5 μM of the specific primers (Additional file 1), 133 μM of each nucleotide (Rapidozym GmbH, Berlin, Germany), 1 x NH_4_ buffer, 2 mM MgCl_2_, and 3 μL of the template.

The PCR conditions for the detection of hemolysin genes included an initial denaturation step at 94 °C for 5 min, followed by 35 cycles at 94 °C for 40 s, annealing at a primer-specific temperature (Additional file 1) for 40 s, elongation at 72 °C for 60 s, and a final elongation at 72 °C for 7 min. The PCR conditions for the detection of *gyrB* were an initial denaturation (5 min, 94 °C), followed by 35 cycles at 94 °C for 30 s, annealing at 60 °C for 30s, elongation at 72 °C for 30 s, and a final elongation at72 °C for 7 min. The PCR conditions for the detection of *adh*, *pgm* and *gdh* followed the protocol of Råsbäck et al. [45]. Amplicons were separated by horizontal electrophoresis using 2% Tris-acetic acid-EDTA (TAE) agarose gels supplemented with 0.5 μg/mL ethidium bromide (Serva Electrophoresis, Heidelberg, Germany) and visualized by UV light. In case of positive signals, the process of RNA purification was repeated.

### Real-time quantitative PCR

Real-Time quantitative (q) PCR was performed on an ABI 7300 Real-time qPCR System (Thermo Fisher Scientific, St. Leon-Rot, Germany) using a qPCR MasterMix for SYBR Green I (Eurogentec, Cologne, Germany). Primers for Real-Time quantification (Additional file 1) were designed with the software Primer Express version 3.0 (Thermo Fisher Scientific, St. Leon-Rot, Germany) to amplify fragments of 62 to 131 bp of the hemolysin and reference genes. Forward and reverse primers were each mixed and stored at − 20 °C in a concentration of 1.0 μM. One reaction mix (25 μL total volume) contained 5 μL of primer mix, 0.75 μL SYBR Green I, 4.25 μL RNase-free water, 12.5 μL 2 x reaction buffer, and 2.5 μL cDNA. Cycling conditions were taken from the manufacturer of the qPCR MasterMix (UNG step 2 min 50 °C, HotGoldStar activation 10 min 95 °C and 40 cycles consisting of 15 s 95 °C and 1 min 60 °C) with an additional melting curve analysis to detect non-specific products like primer-dimers.

Efficiencies of the different qPCR assays were calculated from the slopes of standard curves obtained from 10-fold dilution series of *B. hyodysenteriae* B204 and G423 genomic DNA, respectively. To increase the accuracy, five repetitions of each dilution were performed. Real-time qPCR of cDNA samples (hemolysin genes and reference genes) was performed in triplicate. Outliers were detected with the Grubbs’ test [46] and omitted from further analysis.

### Sequence analysis of gene fragments of whBh strain G423

To verify affiliation of the weakly hemolytic strain G423 to the species *B. hyodysenteriae* we compared the nucleic acid identity of the NADH oxidase (*nox*) gene and the 16S rRNA gene of strain G423 with other *B. hyodysenteriae* strains. Fragments of these genes (*nox* gene, 939 bp; 16S rRNA gene 870 bp) were amplified by PCR and sequenced (LGC Genomics, Berlin, Germany). In a recently published article a disruption of the promoter site of *hlyA* of whBh strains was described [11]. Therefore, the intergenic region of our whBh strain G423 between the *fabG* (ACP reductase) and the hemolysin gene *hlyA* with a length of 1084 bp was also amplified and sequenced. Primers and PCR conditions are listed in Additional file 1.

### Data evaluation and statistical analysis

For each of the three experiments with the shBh and whBh strains, transcription activities of the eight hemolysin genes were determined at different time points at the hemolytic and non-hemolytic stages of growth.

The comparative Ct method (ddCt) was used to analyze differences in gene expression between strongly and weakly hemolytic strains. Statistical analysis was performed with the REST program (Qiagen, Hilden, Germany) which normalizes Ct values of the hemolysin genes to the reference genes and takes different PCR efficiencies into account.

## Supplementary information

**Additional file 1.** Primers and PCR conditions used in this study.

## Data Availability

The datasets analyzed during the current study are available in the NCBI GenBank repository (https://www.ncbi.nlm.nih.gov/genbank/) under accession numbers MT304814 - MT304822.

## Abbreviations

AA: Amino acid
ACP: Acyl-carrier protein
B: *Brachyspira*
BHIF: Brain-heart-infusion broth containing 20% fetal calf serum
BLAST: Basic Local Alignment Search Tool
CBS: Cystathionine beta-synthase
Clp: Caseinolytic protease
CV: Coefficient of variation
GFU: Growth forming unit
*nox*: Gene coding for NADH-oxidase
SD: Swine dysentery
shBh: Strongly hemolytic *Brachyspira hyodysenteriae*
StDv: Standard deviation
TSA: Trypticase soy agar
qPCR: Quantitative polymerase chain reaction
whBh: Weakly hemolytic *Brachyspira hyodysenteriae*
