# Supplementary material for: Differential expression of hemolysin genes in weakly and strongly hemolytic Brachyspira hyodysenteriae strains
Source: BMC Vet Res. 2020 May 29;16:169. doi: 10.1186/s12917-020-02385-5 (PMC7260840; doi:10.1186/s12917-020-02385-5)
Supplement: Supplementary file 1 — Additional file 1. Primers and PCR conditions used in this study. [file 12917_2020_2385_MOESM1_ESM.docx]

| **Additional file 1: Primers and PCR conditions used in this study** | | | | | | | | | |
| --- | --- | --- | --- | --- | --- | --- | --- | --- | --- |
| **Primer** | **Target gene** | **Nucleotide sequence (5' - 3')** | **Amplicon size [bp]** | **Annealing**  **[°C]** | | **Reference**  **strain(s)** | **Reference** | |  |
| 16SF | 16S rRNA | TGCCAGCAGCCGAGGTAA | 870 | 57 | | universal | Chung et al., 2007 | |  |
| 16SR |  | AGRCCCGRGAACGTATTCAC |  |  | | universal | This study | |  |
| ADH_F206 | *adh* (alcohol dehydrogenase) | GAAGTTTAGTAAAAGACTTTAAACC | 552 | 50 | | B204/G423 | Råsbäck et al., 2007 | |  |
| ADH-R757 |  | CTGCTTCAGCAAAAGTTTCAAC |  |  | |  |  | |  |
| GDH-F514 | *gdh* (glutamate dehydrogenase) | GGAGTTGGTGCTAGAGAGAT | 644 | 50 | | B204/G423 | Råsbäck et al., 2007 | |  |
| GDH-R1157 |  | ATCTCTAAAGCAGAAGTAGCA |  |  | |  |  | |  |
| PGM-R1220 | *pgm* (phospho-glucomutase) | CCGTCTTTATCGCGTACATT | 1049 | 50 | | B204/G423 | Råsbäck et al., 2007 | |  |
| PGM-F172 |  | GTTGGTACTAACAGAATGAATA |  |  | |  |  | |  |
| BhtlyAF | *tlyA* | GCAGATCTAAAGCACAGGAT | 527 | 60 | | B204/G423 | Rasbäck et al., 2006 | |  |
| BhtlyAR |  | GCCTTTTGAAACATCACCTC |  |  | | B204/G423 | This study | |  |
| TlyBfor | *tlyB* | TGAAGAGGGAGGCGGACTTA | 613 | 57 | | B204/G423 | This study | |  |
| TlyBrev |  | AGAGCCATCATTAGCATCAACG |  |  | | B204/G423 | This study | |  |
| TlyCfor | *tlyC* | GGTTGTTGATGAATACGGCGG | 252 | 60 | | B204/G423 | This study | |  |
| TlyCrev |  | GGAAGCCTGCCCAAGTATGA |  |  | | B204/G423 | This study | |  |
| BHlyAneu-for | *hlyA* | TCACTGATACAGCTTCTTTCG | 148 | 60 | | B204/G423 | This study | |  |
| BHlyAneu-rev |  | GTATTTAGCAGCATCAGCTAC |  |  | | B204/G423 | This study | |  |
| hlyneu-for | *hemolysin activation protein* gene | CGCCYCGTGTTGATATGGTA | 177 | 57 | | B204/G423 | This study | |  |
| hlyneu-rev |  | TCTTTCCGCCGCCTTTAACA |  |  | | B204/G423 | This study | |  |
| YplQfor | *hemolysin III* gene | ACAGGAGTATATGTTGCGGCT | 223 | 60 | | B204/G423 | This study | |  |
| YplQrev |  | CCCATGGCATAGGAGCTTTACT |  |  | | B204 | This study | |  |
| yplQ-R_423 | *hemolysin III* gene | GCTTTGCTTGGTATTGCAGGA | 208 | 60 | | G423 | This study | |  |
| 1870for | *hemolysin channel protein* gene | CCACGCCATCTGTATCCGAA | 322 | 60 | | B204/G423 | This study | |  |
| 1870rev |  | CGGCAATAGCTGTCTTAGTGC |  |  | | B204/G423 | This study | |  |
| 962for | *hemolysin* gene | AACTTCTCCCCCTTGTCTGC | 355 | 60 | B204/G423 | | This study | |  |
| 962rev |  | CTCAGGCGATACAGTTGCCA |  |  | B204/G423 | | This study | |  |
|  |  |  |  |  |  | |  | |  |
| gyrBfor2 | *gyrB* (gyraseB) | AATATCTGCGGCACAGGCTAGAGA | 319 | 55 | B204/G423 | | This study | |  |
| gyrBrev2 |  | TGAAGCACCTACACCGCATCCTAA |  |  | B204/G423 | | This study | |  |
| BnoxR | *nox* | TAGCYTGCGGTATYGCWCTTTGG | 939 |  | B204/G423 | | Rohde et al., 2002 | |  |
| BnoxF |  | CTTCAGACCAYCCAGTAGAAGCC |  |  | B204/G423 | |  | |  |
| FabG-1-for | *hlyA* promotor site | AAAGGGTACATATGGACTTAAATCTTAAAAATAAAACAGCT | 1048 | 50 | B204/G423 | | This study | |  |
| hlyA-3 |  | CGGAGCTCGAATTCTTATTTTTTATGTTCTTCAATGTAT |  |  | B204/G423 | | This study | |  |
| **RT-PCR Primer** | |  |  |  |  | |  | |  |
| RT-PCR-TlyB-Fo | *tlyB* | AAGGATTCGATAAGAAGTATGGTGCTA | 79 | 60 | B204/G423 | | This study | |  |
| RT-PCR-TlyB-Re |  | TTCGGTACTCACATAATCCTCTATCTCT |  |  | B204/G423 | | This study | |  |
| RT-PCR-TlyA-Fo | *tlyA* | AAAGGCGTTTGTAGAATTTGGAAT | 131 | 60 | B204/G423 | | This study | |  |
| RTPCR-TlyA-Re |  | TGTCCTACATCAAGAGCATAAACTTTTT |  |  | B204/G423 | | This study | |  |
| RTPCR-TlyC-Fo | *tlyC* | TGCTTGAGCAGATTATAGGTGATATTAGA | 78 | 60 | B204/G423 | | This study | |  |
| RTPCR-TlyC-Re |  | GTTCCATCATCATTGCTCTTTATTTC |  |  | B204/G423 | | This study | |  |
| RTPCR-962-Fo | *hemolysin* gene | TGATATTCGGAGAGCTTGTACCTAAA | 72 | 60 | B204 | | This study | |  |
| RTPCR-962-Re |  | GCTGCAACCACTTTTGCTATTCT |  |  | B204/G423 | | This study | |  |
| RTPCR-yplQ-Fo | *hemolysin III* gene | TTTACTTGGTATTGCAGGACTTGTTC | 127 | 60 | B204 | | This study |  |  |
| RTPCR-yplQ-Re |  | CAGGAAAGAAATGATATAAAGAGCTAAATG |  |  | B204 | | This study |  |  |
| RTPCR-hly-Fo | *hemolysin activation protein* gene | CCCGTGTTGATATGGTATGTATTGA | 98 | 60 | B204/G423 | | This study |  |  |
| RTPCR-hly-Re |  | CCTCATAAACAGGAAATCTTGAAAGTC |  |  | B204 | | This study |  |  |
| RTPCR-1870-Fo | *hemolysin channel protein* gene | CATTCCTCCATGCCTAATATCATTAC | 103 | 60 | B204 | | This study |  |  |
| RTPCR-1870-Re |  | CGAATACGCTTCTTAATGTGATGC |  |  | B204/G423 | | This study |  |  |
| RTPCR-HlyA-Fo | *hlyA* | TCACTGATACAGCTTCTTTCGTAGATG | 113 | 60 | B204/G423 | | This study |  |  |
| RTPCR-HlyA-Re |  | CTTGATCTTCTTGAGGAATTTTGATTTC |  |  | B204/G423 | | This study |  |  |
| RTPCR-gyrB-Fo | *gyrB* (gyraseB) | GAGACTCTGCAGGCGGTACTG | 62 | 60 | B204/G423 | | This study |  |  |
| RTPCR-gyrB-Re |  | GCGGTAAAATAGCTTGGAAATGTC |  |  | B204/G423 | | This study |  |  |
| RTPCR423-962F | *hemolysin* gene | GATATTCGGAGAGCTTGTGCCT | 71 | 60 | G423 | | This study |  |  |
| RTPCR423-hlyR | *hemolysin activation protein* gene | CCTCATAAACCGGAAATCTTGAAA | 98 | 60 | G423 | | This study |  |  |
| RTPCR423-1870F | *hemolysin channel protein* gene | CATTCCTCCATGCTTAATATCATTACA | 103 | 60 | G423 | | This study |  |  |
| RTPCR423-yplQF | *hemolysin III* gene | GCTTGGTATTGCAGGACTTGTTC | 116 | 60 | G423 | | This study |  |  |
| RTPCR423-yplQR |  | ATCAGGAAAGAAATGATATAAAGAACTAAATG |  | 60 | G423 | | This study |  |  |
| RTPCR_adhF_ MLST | *adh* (alcohol dehydrogenase) | AATAGTGTAGAAGCTCAGGCTGGATAT | 63 | 60 | B204/G423 | | This study |  |  |
| RTPCR_adhR_ MLST |  | TTTCCATCCGGCAAGCA |  |  | B204/G423 | | This study |  |  |
